# Whole genome analysis of *Rhizopus* species causing rhino-cerebral mucormycosis during the COVID-19 pandemic

**DOI:** 10.3389/fcimb.2023.1251456

**Published:** 2023-10-31

**Authors:** Joy Sarojini Michael, Manigandan Venkatesan, Marilyn Mary Ninan, Dhanalakshmi Solaimalai, Lydia Jennifer Sumanth, Lalee Varghese, Regi Kurien, Rinku Polachirakkal Varghese, George Priya Doss C

**Affiliations:** ^1^ Department of Clinical Microbiology, Christian Medical College, Vellore, Vellore, Tamil Nadu, India; ^2^ Department of Otorhinolaryngology, Christian Medical College, Vellore, Vellore, India; ^3^ Department of Integrative Biology, School of Biosciences and Technology, Vellore Institute of Technology (VIT) University, Vellore, Tamil Nadu, India

**Keywords:** whole genome sequencing, molecular epidemiology, Mucorales, COVID - 19, azole resistance detection

## Abstract

**Introduction:**

Mucormycosis is an acute invasive fungal disease (IFD) seen mainly in immunocompromised hosts and in patients with uncontrolled diabetes. The incidence of mucormycosis increased exponentially in India during the SARS-CoV-2 (henceforth COVID-19) pandemic. Since there was a lack of data on molecular epidemiology of Mucorales causing IFD during and after the COVID-19 pandemic, whole genome analysis of the Rhizopus spp. isolated during this period was studied along with the detection of mutations that are associated with antifungal drug resistance.

**Materials and methods:**

A total of 50 isolates of *Rhizopus* spp. were included in this prospective study, which included 28 from patients with active COVID-19 disease, 9 from patients during the recovery phase, and 13 isolates from COVID-19-negative patients. Whole genome sequencing (WGS) was performed for the isolates, and the *de novo* assembly was done with the Spades assembler. Species identification was done by extracting the ITS gene sequence from each isolate followed by searching Nucleotide BLAST. The phylogenetic trees were made with extracted ITS gene sequences and 12 eukaryotic core marker gene sequences, respectively, to assess the genetic distance between our isolates. Mutations associated with intrinsic drug resistance to fluconazole and voriconazole were analyzed.

**Results:**

All 50 patients presented to the hospital with acute fungal rhinosinusitis. These patients had a mean HbA1c of 11.2%, and a serum ferritin of 546.8 ng/mL. Twenty-five patients had received steroids. By WGS analysis, 62% of the *Rhizopus* species were identified as *R. delemar*. Bayesian analysis of population structure (BAPS) clustering categorized these isolates into five different groups, of which 28 belong to group 3, 9 to group 5, and 8 to group 1. Mutational analysis revealed that in the *CYP51*A gene, 50% of our isolates had frameshift mutations along with 7 synonymous mutations and 46% had only synonymous mutations, whereas in the *CYP51B* gene, 68% had only synonymous mutations and 26% did not have any mutations.

**Conclusion:**

WGS analysis of Mucorales identified during and after the COVID-19 pandemic gives insight into the molecular epidemiology of these isolates in our community and establishes newer mechanisms for intrinsic azole resistance.

## Introduction

Mucorales are common environmental molds that cause mucormycosis. This is an opportunistic fungal infection that is angio-invasive and therefore has high morbidity and mortality. Even though mucormycosis is found worldwide, causative agents are more common in India. Among the order Mucorales, *Rhizopus arrhizus*/*R. oryzae* is the most common species isolated in the laboratory, followed by *Rhizopus microspores, Litchthemia, Cunningamella*, and *Saksena*. Patients with diabetes mellitus, hematological malignancy and chemotherapy, and hematopoietic stem cell transplant, and solid organ transplant recipients on immunosuppressive therapy with iron overload are at risk of developing mucormycosis. The most common clinical presentation is invasive fungal sinusitis or rhino-orbital–cerebral mucormycosis (ROCM), followed by pulmonary, gastrointestinal, cutaneous, and renal mucormycosis.

The delta wave of the pandemic swept through India from May 2021. There was an increase in the incidence of mucormycosis in patients with SARS-CoV-2 (henceforth COVID-19) during this wave around the world, particularly from India. Epidemiological reviews reveal an acute increase in the incidence of ROCM related to COVID-19 infection. Phylogeny of Mucorales isolated during the COVID-19 pandemic has not been studied in India, where many cases were reported, including from our center (Cherian et al., 2022).

Before the COVID-19 pandemic, the death rate for mucormycosis was 50%; however, during the delta wave, fatalities amounted to 85% (Aranjani et al., 2021). Owing to the rise in mucormycosis cases during this wave of the COVID-19 pandemic and its link with fatalities in COVID-19 patients, further studies on mucormycosis are needed particularly to investigate the relationship of Mucorales with COVID-19 patients (Al-Tawfiq et al., 2021).

So far, genotyping of Mucorales has been performed by using the internally transcribed spacer (ITS) region and D1/D2 regions of the 28S rRNA subunit (Nagao et al., 2005), or multilocus sequencing typing of conserved loci (Cendejas-Bueno et al., 2012). These methods do not reflect genome-scale phylogenetic differences adequately or correctly capture strain and species-level diversity. Whole genome sequencing (WGS) has been used in the recent studies to investigate mucormycosis outbreaks. Though it has inherent challenges, WGS analysis will help to understand the biology and pathogenesis of the organism and disease.

Azoles inhibit ergosterol synthesis by interacting with the 14-∝ sterol demethylases, encoded in molds by CYP51 genes. Azole resistance in filamentous fungi are due to overexpression of CYP51A and/or point mutations in the CYP51A gene and overexpression of efflux pumps. Macedo et al. in 2018 describe that in *Rhizopus oryzae*, CYP51 genes are uniquely responsible for intrinsic resistance to short-tailed triazoles such as voriconazole and fluconazole (Macedo et al., 2018).

Therefore, in this study, we performed WGS on 50 isolates of *Rhizopus* spp. isolated during the delta wave of the COVID-19 pandemic from COVID-positive, -recovered, and -negative patients. We wanted to ascertain the phylogenetic relationship among the isolates in these three groups and to study whether evolutionary clusters and the presence of mutations in the CYP51 genes played a role in the severity of the disease in COVID-19 patients.

## Materials and methods

### Ethics

This study was approved by the Christian Medical College, Vellore Institutional Review board and Ethics committee (IRB no. 14007).

### Study population and sample collection

This was a prospective study done at Christian Medical College Vellore, a large tertiary care teaching hospital that saw many patients with COVID-19-associated mucormycosis. Consecutive clinical isolates of *Rhizopus arrhizus* were cultured from patients with ROCM during the delta wave of the COVID-19 pandemic between March 2021 and December 2021 to collect isolates from post-COVID-19 and COVID-19-negative patients. All isolates were retrieved from patients presenting with a rhino-orbital cerebral sinusitis and were from the sinus tissue; some had extensions into the brain and some into the bone and orbit. The sinus tissue samples obtained from these patients were minced with sterile scissors in a sterile petri dish. The presence of sparsely septate broad, irregular hyphae branching at obtuse angles on microscopic calcofluor white microscopic preparation was identified, and it was cultured on Sabourauds Dextrose Agar with and without antibiotics as per standard laboratory procedure. Characteristic features on culture and microscopy identified the cultures. COVID-19 testing at our center was carried out by the Altona Realstar SARS-CoV-2 RTPCR kit and the Cepheid Xpert Xpress SARS-CoV-2 assay. Of the 50 isolates collected during this period, 28 belonged to patients with active COVID-19 disease (within 3 weeks of RT-PCR positivity), 9 were from patients in the recovery phase (after 3 weeks of RT-PCR positivity), and 13 isolates were from COVID-19-negative patients (negative RT-PCR test).

### DNA extraction

Mucorale isolates were stored at room temperature and subcultured onto Sabouraud Dextrose Agar before processing for DNA extraction. Once grown on Sabouraud Dextrose Agar, they underwent Genomic DNA extraction using the QIAamp DNA Mini Kit (QIAGEN, Hilden, Germany) per the manufacturer’s instructions. Good-quantity and -quality DNA was selected, and WGS was further carried out. The isolated DNA was quantified using a QubitTM 3 Fluorometer (Thermo Fisher Scientific) and a minimum of 0.3 ng/µL DNA concentration was required to perform WGS. DNA quality was verified by running agarose gel electrophoresis to detect nucleic acid degradation. Extracted DNA was stored at −20°C until further use.

### Whole genome sequencing

KAPA HyperPrep Kit (Roche) was used to prepare Illumina sequencing libraries according to the manufacturer’s instructions. After preparing the DNA sample libraries, they were purified with Ampure XP Reagent (Beckman Coulter), quantified with 5300 Fragment Analyzer (Agilent), and uniquely barcoded multiple samples libraries were normalized together to be sequenced equally and simultaneously in a single run. Then, the libraries were sequenced with a 2×150-bp paired-end reads chemistry on the Illumina NovaSeq platform as per the manufacturer’s instructions, resulting in an average of 100× coverage of the whole genome per isolate for all samples.

### Genome assembly

Sequence reads were trimmed to remove poor-quality bases using Trimmomatic (v0.39) followed by *de novo* assembly with Spades (v3.14.1) with the following k-mer lengths: 27, 33, 55, and 75.

### Species tree generation based on ITS gene

**Figure 1:**
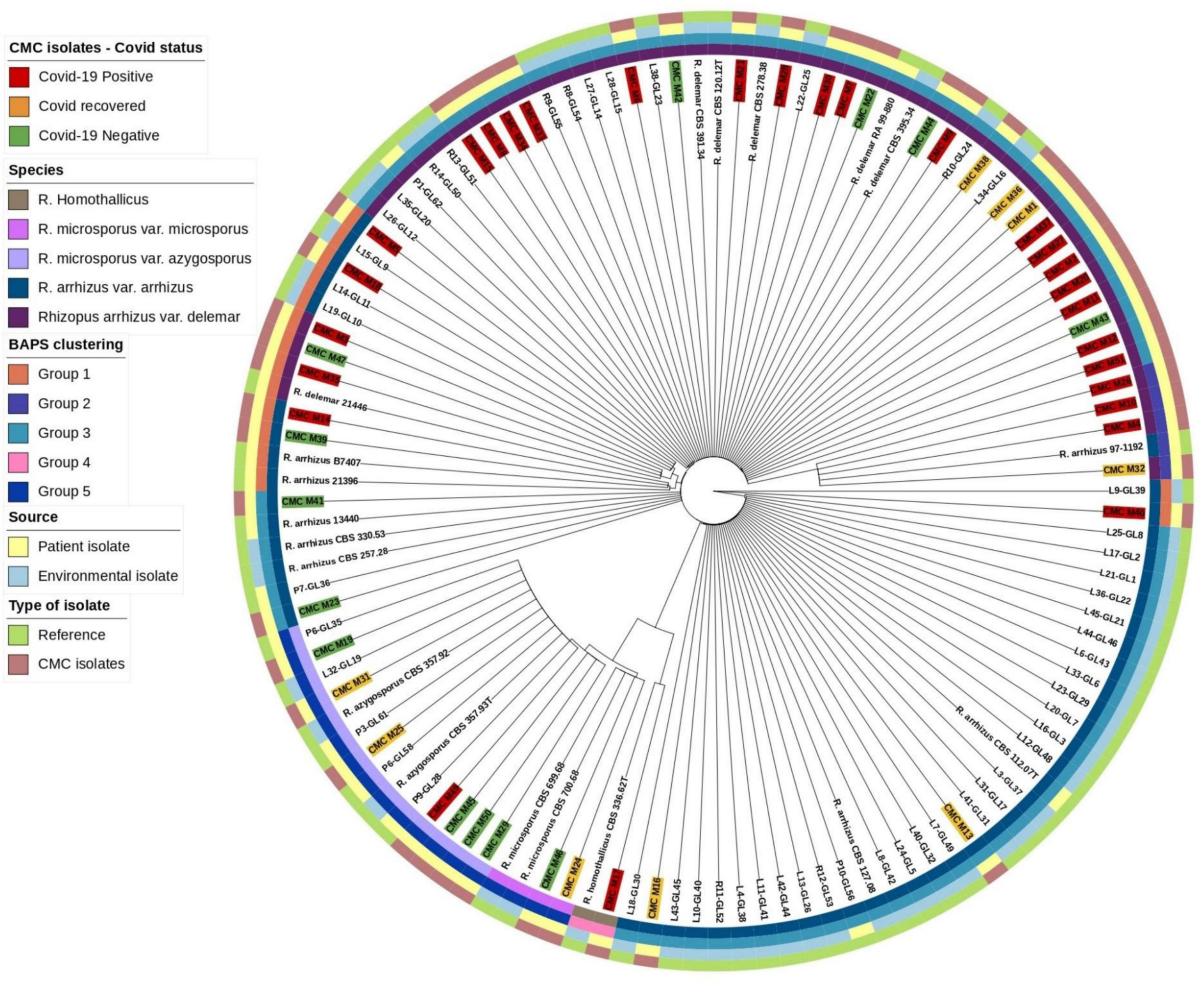
Phylogenetic tree based on ITS gene.

Mucorales, in comparison with other genetic targets like 18S and D1/D2 of the 28S gene ITS region, shows higher species-specific variability and may further discriminate species in *Rhizopus* species (Nagao et al., 2005). Abe et al. also describe better clustering of isolates using ITS region sequencing (Abe et al., 2006). Based on this, ITS gene sequences were extracted from our isolates and reference isolates genome with the BLASTN 2.12.0+ tool and the combined sequence of ITS1-5.8S-ITS2 genes were searched as a query in the Nucleotide BLAST database (https://blast.ncbi.nlm.nih.gov/) for species identification. Genetic clustering analysis for our isolates along with reference isolates was done with RhierBAPS 1.1.4 (Tonkin-Hill et al., 2018). ITS gene sequence multiple alignment was done using Geneious software (https://www.geneious.com/) with Clustal Omega v1.2.3 (Sievers and Higgins, 2018) for our isolates along with the reference isolates, and species tree was generated with RAxML 8.2.11 with the following parameters: GTRGAMMA nucleotide model, Rapid Bootstrapping and a search for best-scoring maximum likelihood tree, 100 bootstrap replicates with parsimony random seed 100, followed by visualization and annotation using iTOL (https://itol.embl.de/) (**Figure 1**).

Based on work from Macedo et al. (2018), two types of CYP51 genes were identified in the *R. oryzae* genome, and they were classified as CYP51A and CYP51B (Macedo et al., 2018). *R. oryzae* ATCC 11886 CYP51A and CYP51B gene sequences were downloaded from the NCBI database and blasted with our isolate genome with the help of the BLASTN 2.12.0+ tool; conversion of the resulting nucleotide sequence to protein sequence followed by multiple alignment with reference protein sequence was done using Geneious software (https://www.geneious.com/) with Clustal Omega v1.2.3 and studied for mutations.

### Phylogenetic tree construction using eukaryotic reference marker genes

**Figure 2:**
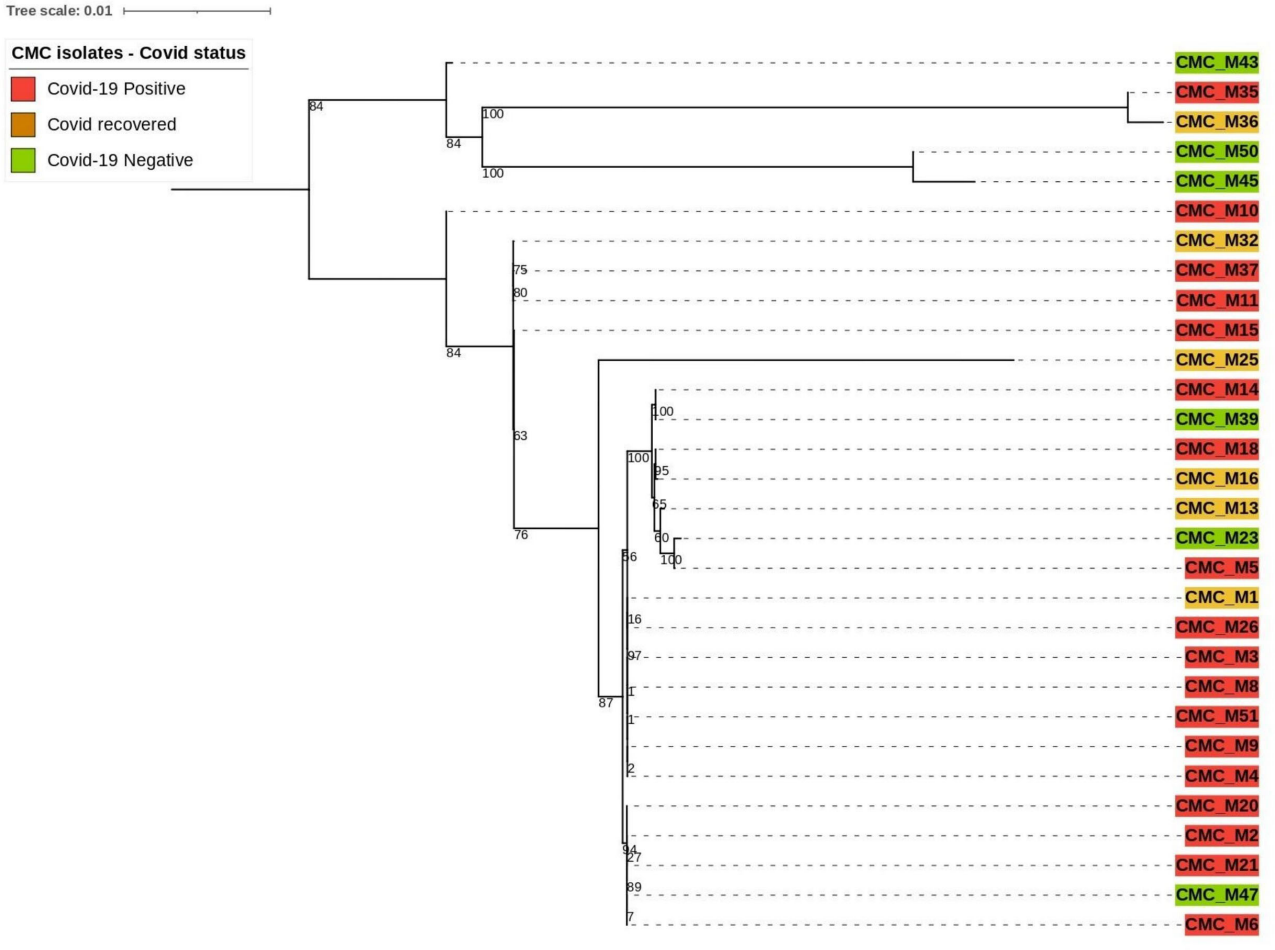
Phylogenetic relatedness based on eukaryotic reference markers.

Our clinical isolate-assembled genomes were analyzed with the Benchmarking Universal Single‐Copy Orthologs (BUSCO v5.4.4) tool to look for orthologous groups specific to the fungi_odb10 lineage using Augustus (v3.3) as described by Simao et al. and Stanke et al (Simão et al., 2015). (Stanke et al., 2006) and the following parameter: “*Rhizopus oryzae*” species was selected for genome assessment mode and protein-coding genes were predicted. Predicted protein sequences were extracted from the AUGUSTUS output. The PhyloSift reference marker genes for eukaryotes as described by Darling et al. (2014) were downloaded and concatenated into one combined file for query and then searched against our isolates that predicted protein sequences (Nguyen et al., 2020). From the 33 reference marker genes found to be conserved among all eukaryotic organisms, only 12 of them were present with complete sequence across 30 of our clinical isolate genomes; thus, they were utilized to compare the phylogenetic relatedness between those isolates (**Figure 2**). Those are 14_3_3, Actin_noOuts, Atub_noOuts, Btub_noOuts, enolase, gamma_noOuts, hsp70, hsp70cyt, hsp70er, Rps23a_noOuts, TFIIH, and U5. Nucleotide sequences of these marker genes from our isolates were aligned using Geneious software (https://www.geneious.com/) with Clustal Omega v1.2.3 as described by Sievers and Higgins et al (Sievers and Higgins, 2018). to make a combined multiple alignment file followed by Phylogenetic tree construction with RAxML 8.2.11 (Stamatakis, 2014) with the following parameters: GTRGAMMA nucleotide model, Rapid Bootstrapping and search for best-scoring maximum likelihood tree, 100 bootstraps replicates with parsimony random seed 100, followed by visualization and annotation using iTOL (https://itol.embl.de/).

### Quality controls

Negative water controls were used for the extraction and subsequent WGS. PhiX controls were used for library preparation. All isolates were blasted with reference isolates. ATCC strains were not used as positive controls.

## Results

### Cohort description

Fifty clinical isolates were cultured from patients diagnosed with ROCM during the study period. The study population included 38 male and 12 female patients with a mean age of 50.28 (28–81) years. Most patients were from Tamil Nadu (34), followed by the adjoining state, Andhra Pradesh (Anand et al., 2022). As shown in **Table 1**, 28 patients were COVID-19 positive, 13 were COVID-19 negative, and 9 were COVID-19 recovered, and the three groups were compared using ANOVA. All *-*values <0.05 were considered significant.

**Table 1:** Clinical and laboratory findings of the patients with mucormycosis.

	Total (*N* = 50)	COVID positive (*N* = 28)	COVID recovered (*N* = 9)	COVID negative (*N* = 13)	*p*-value
**Age**					0.938
Mean ± SD	50.28 ± 12.65	50.50 ± 14.18	48.89 ± 9.78	50.77 ± 12.20	
Range	28-81	28-81	37-61	33-76	
**Gender**					0.052
Male (%)	38 (76)	19 (67.9)	9 (100.0)	10 (76.9)	
Female (%)	12 (24)	9 (32.1)	0	3 (23.1)	
Comorbidities					
DM	50 (100)	28 (100)	9 (100)	13 (100)	–
DKA	7 (14)	6 (21.4)	1 (11.1)	0	0.077
**Duration of symptoms (days)**					0.950
Mean ± SD	10.5 ± 25.04	11.21 ± 33.26	11.11 ± 9.03	8.54 ± 7.90	
Range	1–180	1–180	4–28	2–25	
**Species**					0.057
*Rhizopus arrhizus* (%)	44 (88)	27 (96.4)	8 (88.9)	9 (69.2)	
*Rhizopus microsporus* (%)	6 (12)	1 (3.6)	1 (11.1)	4 (30.8)	
**Prior hospital admission** (%)	28 (56)	17 (60.7)	8 (88.9)	3 (23.1)	0.005
**Steroids given** (%)	25 (50)	17 (60.7)	6 (66.7)	2 (15.4)	0.010
Disease extent (%)					
Nose and PNS	50 (100)	28 (100)	9 (100)	13 (100)	–
Orbit	23 (46)	13 (46.4)	2 (22.2)	8 (61.5)	0.177
Palate	18 (36)	5 (17.9)	5 (55.6)	8 (61.5)	0.009
Intracranial	10 (20)	5 (17.9)	1 (11.1)	4 (30.8)	0.488
Blood parameters					
**HbA1C**					0.836
Mean ± SD	11.2 ± 2.35	11.32 ± 2.39	11.34 ± 2.26	11.80 ± 2.54	
Range	5.9–15.6	6.2–>14	8.2–14.9	5.9–>14	
**Serum ferritin**					
Mean ± SD	546.81 ± 424.64	578.66 ± 446.12	427.86 ± 425.6	567.62 ± 419.24	
Range	120.9–1,909.6	123.1–1,909.6	133.4–1,433.8	120.9–1,423.4	
**HPE (%)**					0.639
AIFS	29 (58)	16 (57.1)	4 (44.4)	9 (69.2)	
AIFS + CGFS	20 (40)	11 (39.3)	5 (55.6)	4 (30.8)	
No biopsy	1 (2)	1 (3.6)	0	0	
**Osteomyelitis (%)**					0.082
Nil	32 (64)	18 (64.3)	4 (44.4)	10 (76.9)	
At initial presentation	4 (8)	1 (3.6)	3 (33.3)	0	
Late presentation	14 (28)	9 (32.1)	2 (22.2)	3 (23.1)	
**Outcome (%)**					0.187
Alive with no clin/radio disease	30 (60)	17 (60.7)	7 (77.8)	6 (46.2)	
Alive with radio+ clinical	9 (18)	4 (14.3)	2 (22.2)	3 (23.1)	
Alive with clinical	1 (2)	1 (3.6)	0	0	
Dead	8 (16)	6 (21.4)	0	2 (15.4)	
LFU	2 (4)	0	0	2 (15.4)	

All tests were compared using ANOVA, p < 0.05 was taken as significant.

The mean duration of symptoms was 10.5 days. A total of 28 patients gave a history of prior hospital admission, and 25 had received steroids. All patients had sinonasal involvement. Forty-six percent had additional orbital involvement, while the palatal and intracranial extension was seen in 36% and 20%, respectively. Extra paranasal sinus involvement was seen predominantly among the COVID-negative patients. Four patients had bony involvement at presentation, while another 14 showed late bony changes.

Among the COVID-19-positive patients, 14 tested positive at admission, while the rest presented within 3 weeks of testing positive. All patients in the cohort had diabetes. Of the seven diabetic patients who presented with ketoacidosis, six were COVID-19 positive. The mean HbA1c was 11.2%, and serum ferritin was 546.8 ng/mL in the cohort. Among the 50 patients with AIFS, 20 patients had associated CGFS.

On follow-up, 40 (80%) patients were alive, among whom 30 had no clinical or radiological evidence of disease, 1 patient had the residual disease, while 9 patients though clinically and endoscopically normal, had radiological changes. Eight (16%) patients had expired, and two were lost to follow-up.

### Phylogenetic assessment

Based on the ITS gene (**Figure 1**), Bayesian analysis of population structure (BAPS) clustering algorithm clustered 50 isolates from CMC and reference isolates into five groups. Group 1 comprises eight of our clinical isolates identified as *R. arrhizus* (*n* = 3) and *R. delemar* (*n* = 5) species closely clustered to the clinical reference strains *R. arrhizus* (B7407 and 21396) and *R. delemar* (21446). Group 2 contains four of our clinical isolates; all were identified as *R. delemar; R. arrhizus* 97-1192 strain shows close relatedness with these groups. Thirty-four clinical isolates were found to be clustered in group 3, 28 CMC isolates clustered with six reference clinical strains, *R. arrhizus* CMC strains (Nagao et al., 2005) were closely related to *R. arrhizus* (13440 and CBS_112.07T), and, similarly, *R. delemar* CMC strains (24) were closely associated with *R. delemar* (RA 99-880). Group 4 consists of one clinical isolate identified as *R. homothallicus* species. Group 5 was divided into two subgroups: 2 clinical isolates identified as *R. microsporus* and 11 clinical isolates (7 isolates from CMC clustered with 4 reference strains) identified as *R. azygosporous*. **Table 2** summarizes the BAPS groups stratified by COVID-19-negative, COVID-19-positive, and COVID-19 recovered patients.

**Table 2:** Distribution of the Mucorales into the various BAPS clusters.

BAP clusters (*n* = 5)	COVID-19-positive patient isolates	COVID-19-recovered patient isolates	COVID-19-negative patient isolates	Total number of patient isolates observed in each group	Species identification by BAPS clustering
Group 1	6	0	2	8	*R. arrhizus* (3), *R. delemar* (5)
Group 2	3	1	0	4	*R. delemar*
Group 3	17	5	6	28	*R. arrhizus strains* 13440 and CBS_112.07T (4), *R. delemar* (28)
Group 4	1	0	0	1	*R. homothallicus*
Group 5	1	3	5	9	*R. microsporus* (2), *R. azygosporous* (7)

BAPS, Bayesian analysis of population structure.

Based on the mentioned eukaryotic reference marker genes, 30 isolates’ genomes were utilized for building the phylogenetic tree to explain the genetic relatedness. This divided 30 clinical CMC isolates into two major branches. The top branch consists of three COVID-19-negative isolates, a COVID-19-positive isolate, and a COVID-19-recovered isolate, and the lower branch consists of 25 isolates composed of 17 COVID-19-positive, 3 COVID-19-negative, and 5 COVID-19-recovered patient isolates.

### Phylogenetic tree inference

In our 50 clinical isolates (**Figure 1**), 31 isolates were identified as *R. delemar*, which includes 22 isolates from COVID-19-positive patients, 4 isolates from COVID-19-recovered patients, and 5 isolates from COVID-19-negative patients; 9 isolates identified as *R.* arrhizus, consisting of 4 COVID-19-positive, 2 COVID-19-recovered, and 3 COVID-19-negative patient isolates; 7 isolates were identified as *R. azygosporous*, comprising 1 COVID-19-positive, 2 COVID-19-recovered, and 4 COVID-19-negative patient isolates; 2 isolates were identified as *R. microsporus* species, which contained one COVID-19-recovered and COVID-19-negative patient; and 1 COVID-19-positive patient isolate belonged to the *R. homothallicus* species. From the BAPS clustering, we observed that 28 of our patient isolates belong to group 3; 17 of them were isolated from COVID-19-positive, 5 from COVID-19-recovered, and 6 from COVID-19-negative patients. All the COVID-19-positive isolates in group 3 were identified as *R. delemar.*

### Summary of mutations

**Figure 3:**
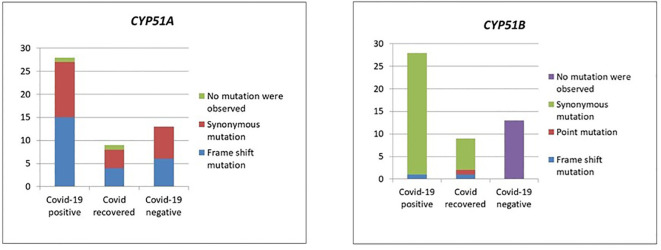
Mutations in the CYP51A and CYP51B genes.

Except in CMC_M21 and CMC_M36 isolates, mutations were observed in the CYP51A gene sequences in all 50 isolates with T nucleotide insertion at the 144 and 145 nt, T nucleotide insertion at 263 nt, C330T, C339T, C375T, G798A, A831G, T1008C, and T1542C. In the mutations observed, only the insertion mutations altered the protein sequence, which leads to frameshift since all the other mutations were synonymous. We have also observed mutations in CYP51B at C75T, T129C, insertion of A at the base of 255 nt, C378T, C575T, G1708A, T1144C, and A1485C. In CMC_M1, C575T and G1708A nucleotide changes were observed that lead to protein sequence change P192L and V570I, respectively; all the other mutations observed in this gene sequence of our isolates were synonymous. Insertion mutations were observed in the isolates CMC_M21 and CMC_M36 at CY51B, which lead to the frameshift of protein sequence; these isolates also did not have any mutations at CY51A. **Figure 3** summarizes the findings.

## Discussion

Mucormycosis is an acute invasive disease-causing rhino-cerebral mucormycosis in patients with diabetes mellitus and immunocompromised patients such as bone marrow or organ transplants. During the delta wave of the COVID-19 pandemic, there were increased cases of mucormycosis among patients with COVID-19 infection. This is the first study looking at the molecular epidemiology of the Mucorales during and after the pandemic.

The molecular epidemiology of mucormycosis has been studied to investigate outbreaks among solid organ transplant patients and compare it with the environmental isolates (Simão et al., 2015). Here in our study, we used WGS to compare the molecular epidemiology and relatedness of the Mucorales in COVID-19-positive, COVID-19-recovered, and COVID-19-negative patients.

As described by our center as well as a study done across India, the most common risk factor for mucormycosis among COVID-19 patients was diabetes mellitus with 21% among the COVID-19-positive patients having diabetes ketoacidosis. The high serum glucose and ferritin levels secondary to uncontrolled diabetes mellitus and ketoacidosis in a hypoxic acidic medium, in combination with COVID-19-induced decreased phagocytosis, stimulated an ideal state for the escalation of mucormycosis. Similar to the case–control investigation done in 11 hospitals across India, the common presentation in our center was rhino-cerebral mucormycosis, both during and after the pandemic (Anand et al., 2022).

### Phylogenetic tree

WGS-employed phylogenetic analysis results in a higher resolution in establishing association among isolates. The phylogenetic tree constructed using ITS1 gene sequences (**Figure 1**) from the clinical and reference isolates was clustered into five groups. Group 3 consisted of the highest number of clinical isolates, of which COVID-19-positive individuals were found at a higher abundance in the group (**Table 2**). Groups 3 and 5 showed a dichotomous tree, separated into new groups based on the subspecies type. Using a synoptic approach, clinical isolates were linked to environmental isolates. Three isolates from group 1, group 3, group 4, and group 5 were closely associated with environmental isolates, indicating the involvement of strains from hospital or the surrounding community. In the ITS-based tree, most of the isolates belonged to *R. delemar* species, indicating it to be the responsible strain that had been widely distributed. The top branch homology between the clinical isolates is in the core genome tree. Most of the COVID-19-positive patients were categorized into the *R. delemar* group. A close clustering can also be observed between the COVID-19-positive isolates and the COVID-19-recovered isolates; a close association was also observed between the *R. delemar* and *R. arrhizus* species, indicating a genetic relatedness between them. As observed in several studies, our study finds it tenable that various species are involved in the incidence of mucormycosis outbreaks instead of a solitary strain (Neblett Fanfair et al., 2012; Garcia-Hermoso et al., 2018). Thus, in this study, we were able to ascertain that the *Rhizopus* spp. causing rhino-cerebral mucormycosis during the COVID-19 pandemic were as diverse as the strains after the epidemic. Though we found increased number of group 3 isolates in COVID-19-positive patients, there was no particular clustering of the difference groups, indicating that there was not a common source for the increased surge of cases during the pandemic.

### Mutation analysis

Mutation analysis (**Figure 3**) revealed that in the CYP51A gene, a T nucleotide insertion was observed at the base of 144, 145, and 263 on the sequence that leads to a frameshift of protein translation in 25 isolates (15 COVID-19-positive, 4 COVID-19-recovered, and 6 COVID-19-negative patients).

The following nucleotide changes were also observed along with frameshift mutation in the same isolates: C330T, C339T, C375T, G798A, A831G, T1008C, and T1542C. The A831G, T1008C, C1005T, and C1162T mutations were present without the frameshift mutation in another 23 isolates (from 12 COVID-19-positive, 4 COVID-19-recovered, and 7 COVID-19-negative patients). Since these were synonymous mutations, protein sequences were not altered. Two isolates, CMC_M21 and CMC_M36, from a COVID-positive and a COVID-recovered patient, respectively, did not have any nucleotide changes in the *CYP51A* gene sequence. These two were also the only isolates that had a nucleotide A insertion in the *CYP51B* gene sequence, which led to a frameshift mutation, present along with the following synonymous mutations: C75T, C378T, and T1144C. Like CYP51A, at *CYP51B*, some synonymous mutations, namely, C75T, T129C, C378T, and A1485C, were present without any frameshift mutation in 34 isolates comprising 27 COVID-19-positive and 7 COVID-19-recovered patient isolates, but unlike CYP51A, one COVID-19-recovered patient isolate (CMC_M1) had two point mutations in the CYP51B gene—C575T and G1708A—that led to protein sequence change P192L and V570I, respectively. Of the COVID-19 patients, 13 COVID-19-negative patient isolates did not have any of these mutations in the CYP51B sequence, whereas all isolates had mutations in the CYP51A gene sequence. The CYP51A gene is uniquely responsible for the intrinsic azole resistance phenotype and not CYP51B; CYP51B gene mutations were rarely reported in studies done on fungal isolates. Similarly, we also did not find any mutations in 13 isolates from COVID-negative patients. Further studies are required to understand the CYP51B gene functions and its mutations’ involvement in the azole resistance.

Azoles act intracellularly by binding and inhibiting a key enzyme in the ergosterol pathway, lanosterol 14-αdemethylase, a cytochrome P450 enzyme (named ERG11 or CYP51A depending on the fungus) (Jensen, 2016). The mechanism of intrinsic azole intrinsic resistance in Mucorales includes overexpression and/or point mutations in the CYP51A gene. Macedo et al. (2018) analyzed the role of the CYP51A gene and its mutations causing intrinsic resistance to voriconazole and fluconazole in Mucorales. They have demonstrated that the gene sequence of CYP51A can be solely responsible for this intrinsic resistance. They hypothesized that azole resistance in Mucorales would occur because of Y132F and/or F145M substitutions in CYP51A, based on *C. albicans* Erg11p amino acid sequence numbering. In our study, we have not found any point mutation as mentioned above. Limited literature available shows that CYP51 mutations are associated with resistance to voriconazole and fluconazole but not posaconazole or itraconazole (Chau et al., 2006). In addition to synonymous mutations in the CYP51A gene sequence, 25 isolates had a frameshift mutation in the CYP51A gene sequence due to the insertion of T nucleotide at the base of 144, 145, and 263 that leads to alteration in the protein translation. These frameshift mutations were unique and have not been described by Macedo et al. (2018). We have also analyzed the CYP51B gene sequence of our isolates and inferred the results. Based on our observation, we have found that two point mutations, namely, P192L and V570I, in one isolate and one insertion mutation, nucleotide A at the base of 255, led to frameshift of protein translation in two isolates in the case of the CYP51B gene along with some synonymous mutations. Further analysis is required to evaluate the importance of these mutations. The significance of these mutations can be ascertained only by performing *in vitro* and *in vivo* antifungal drug susceptibility testing of these isolates and comparing with the clinical outcome of the patients.

One of the main challenges is that mutations and phylogenetic analysis of Mucorales by WGS has been applied infrequently in studies of mucormycosis because the mucormycete genomes are complex and there are very few scaffolds for assembling the genomes. There are only few clinical isolates in existing databases to compare our isolates for strain relatedness using SNP differences as is being used for other microorganisms.

The main limitation of the study was that the sample size was small and involved those collected over a short period of time during the acute phase of the COVID-19 pandemic and after COVID-19. In non-COVID-19 times, we see mucormycosis in different clinical spectra (Manesh et al., 2019). All patients included in this study during the COVID pandemic had diabetes mellitus as a risk factor. No other immunocompromised status was detected in any of them. Therefore, one of the limitations of the study is that the results of the study may not be representative of patients with other immunocompromised conditions. We also did not perform environmental sampling to look for Mucorales in the community. Thus, we were not able to demonstrate the source of these organisms or demonstrate any particular clustering of clades of *Rhizopus* spp. We also could not compare these genotypic data to phenotypic antifungal susceptibility for mucormycosis.

In summary, WGS on Mucorales is essential to ascertain the phylogenetic relationships among isolates in a hospital or in a community and compare it elsewhere in the country or globally. It also allows for analysis of resistance and virulence markers, which can unravel the biology and pathogenesis of these species. This study emphasizes the need for larger studies to comprehend the molecular epidemiology of these organisms and also the need to standardize WGS-based typing methods for Mucorales and to validate interpretive criteria for strain relatedness.

## Data availability statement

The data presented in this study has been deposited and made publicly available in NCBI, https://www.ncbi.nlm.nih.gov/sra/PRJNA993381. Any further enquiries can be directed to the corresponding author.

## Ethics statement

This study was approved by the Christian Medical College, Vellore Institutional Review board and Ethics committee (IRB no. 14007).

## Author contributions

JM: Design, execution of the study. Analysis of the data, write up and finalisation of the article; MV: WGS and Analysis; MN: Analysis and write up; DS: Collection of Rhizopus isolates, laboratory and clinical data mining; LS: Collection of Rhizopus isolates, laboratory and clinical data mining; LV: Clinical management of patients; RK: Clinical management of patients; RV: Bioinformatics analysis; GC: Bioinformatics analysis. All authors contributed to the article and approved the submitted version.
